# Eosinophilic pustular folliculitis (EPF) in a patient with HIV infection

**DOI:** 10.1007/s15010-020-01543-z

**Published:** 2020-11-25

**Authors:** T. Kanaki, E. Hadaschik, S. Esser, Stefanie Sammet

**Affiliations:** Department of Dermatology, University Hospital Essen, University of Duisburg-Essen, Hufelandstr. 55, 45122 Essen, Germany

## Abstract

Eosinophilic pustular folliculitis is a chronic, recurrent dermatosis, of unknown etiology, which is histologically characterized by folliculotropic inflammatory infiltrates with admixed eosinophils in the dermis. It has often presented with immunosuppression and especially with HIV-Infection. In the HAART-era, eosinophilic pustular folliculitis has become a rarity. It is often being misdiagnosed as acne vulgaris, rosacea, bacterial folliculitis, dermatomycosis and seborrheic dermatitis. The treatment of this disease may be difficult.

A 51-year-old Ghanaian, female patient presented in our outpatient department of venereology in January 2020 with a recently diagnosed HIV infection, to continue her treatment. The patient had pruritic skin eruption on her face and upper buttock that had appeared 6 months prior to presentation in our department. Inspection revealed multiple disseminated papules and plaques on her face and on her back with post-inflammatory hyperpigmentation. No scratch excoriations were noticed on the face of the patient (Figs. 1, 2). On the back, the patient had multiple excoriations revealing a moderate-to-severe pruritus. The patient was being treated until then for acne vulgaris with metronidazole gel and azelaic acid 15% gel and showed no improvement.

**Fig. 1:**
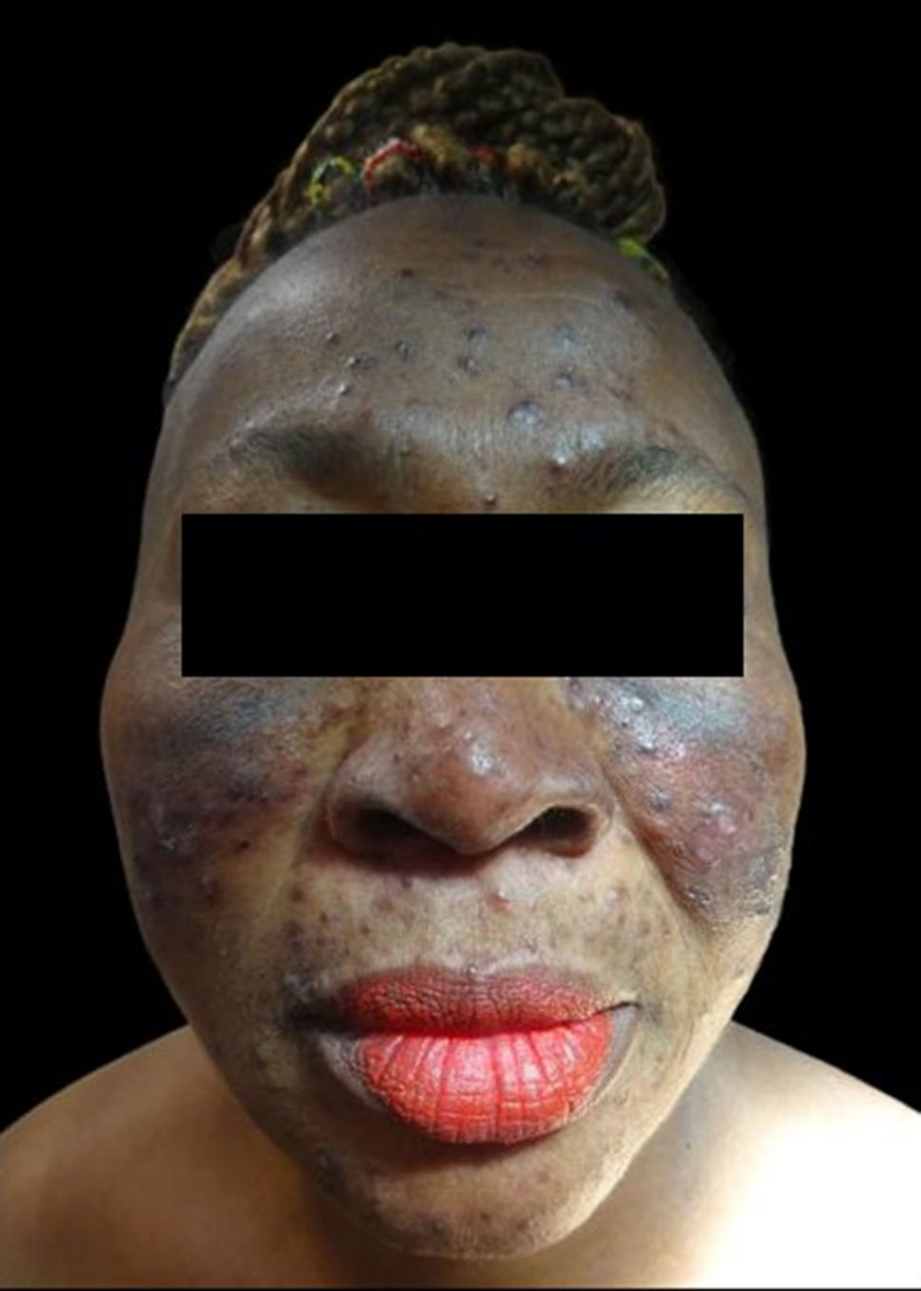
Multiple, disseminated papules and post-inflammatory hyperpigmentations on the face of the patient

**Fig. 2:**
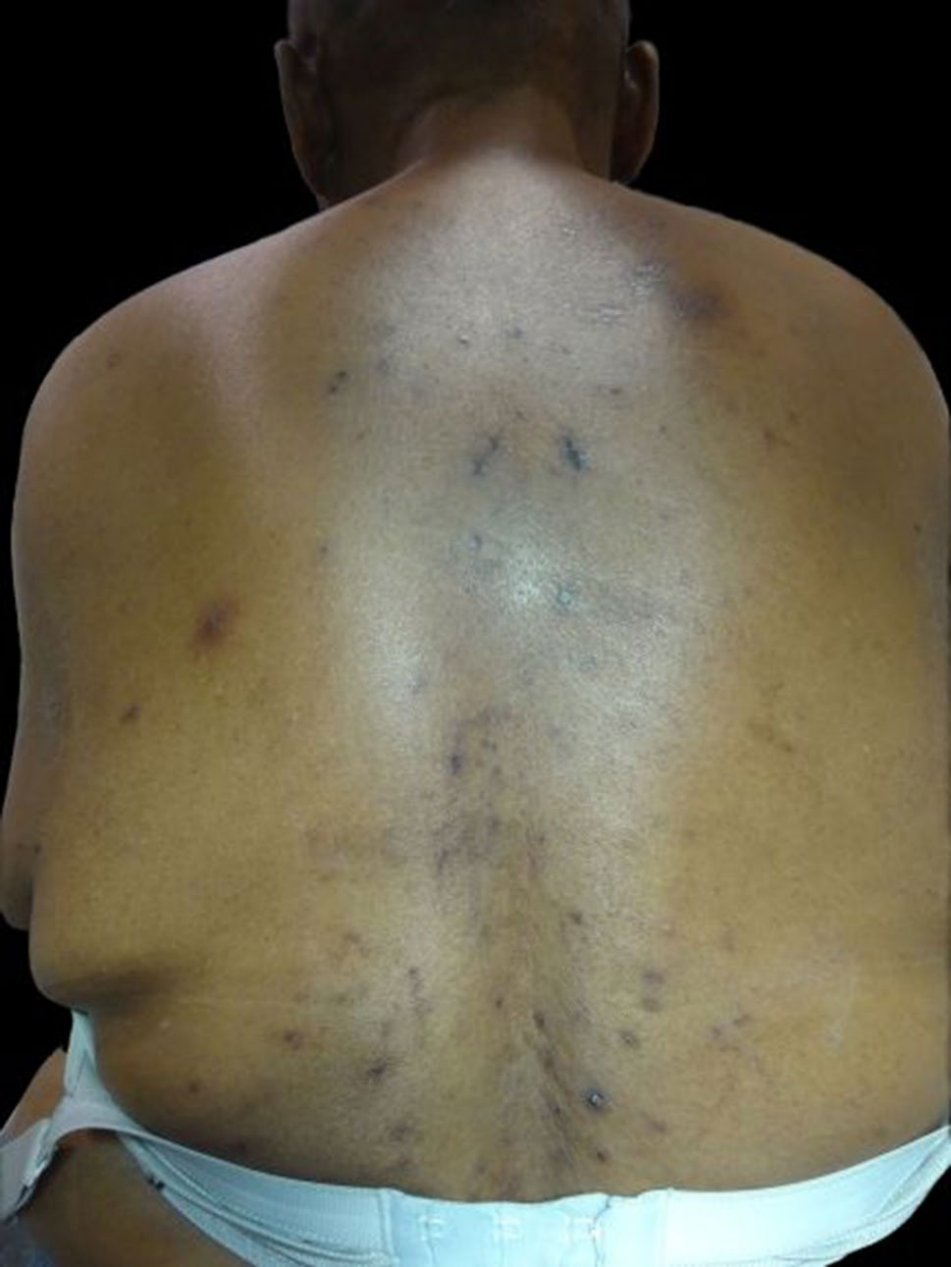
Multiple, disseminated papules, post-inflammatory hyperpigmentations and multiple excoriations on the back of the patient

The patient lives in Germany since June 2013. In November 2019, she was diagnosed with the HIV infection and since then was being treated with Emtricitabin 200 mg/Tenofovir Alafenamid 25 mg once per day and Raltegravir 400 mg twice per day. Her CD4 ( +)-T-lymphocyte count was 101/µL (604–1188/µL), virus load was 1.5 million copies. Additional laboratory findings of the patient were the following: leucocyte count: 4, 90/nL (3, 6–9, 2/nL), blood eosinophilia 1% (2–4%), C-reactive protein 0.5 mg/dL (< 0.5 mg/dL), early secretory antigen target-6 (ESAT-6) 32 (< 6) and culture filtrate protein-10 (CFP-10) 37 < 6.

The possible differential diagnoses were acne vulgaris, rosacea, bacterial folliculitis, dermatomycosis, seborrheic dermatitis, cutaneous T-cell lymphoma follicular mucinosis and eosinophilic pustular folliculitis. Acne vulgaris is most commonly localized on the face, chest and back. It appears normally by adolescents between 14 and 18 years old. The characteristic lesion is comedone, which was absent in our case. Rosacea is a common disease, which is characterized alone or in combination, by central facial erythema, symmetric flushing, stinging sensation, inflammatory lesions (papules and pustules), telangiectasias, and phymatous changes. It is typically located on the face. Extrafacial localization is very rare. For bacterial folliculitis, no purulent lesions were observed. Dermatomycosis can occur on every part of the skin. Typical findings are circumscribed areas of alopecia and inflammation, which begin as small papules that spread peripherally in a scaly inflamed ring. Seborrheic dermatitis is a common skin condition with characteristic scaling, erythema and itching, commonly localized on the scalp, face, chest, back, axilla and groin. Scaling and erythema were absent in this case. A cutaneous T-cell lymphoma is normally presented with nodules 2.4–7.0 cm on the upper trunk and especially on the upper extremities. Typically, it is located on only one body region. The nodules may present a central ulceration. Follicular mucinosis is a rare skin disorder. It may appear on children and young adults but also on adults after the age of forty. The lesions may be localized on the skin of the face, capillitium, trunk and proximal extremities. Folliculotropic papules and alopecia are characteristics. There are two forms: the primary and the secondary form. Accordingly, the presence of an underlying disease, such as folliculotropic mycosis fungoides (a rare type of T-cell lymphoma), should be excluded. On our patient, no alopecia was observed.

Biopsies of the skin lesions were performed on the left cheek and back of the patient to exclude a cutaneous T-cell lymphoma and a follicular mucinosis.

The histologic findings revealed an EPF (Figs. 3, 4).

**Fig. 3:**
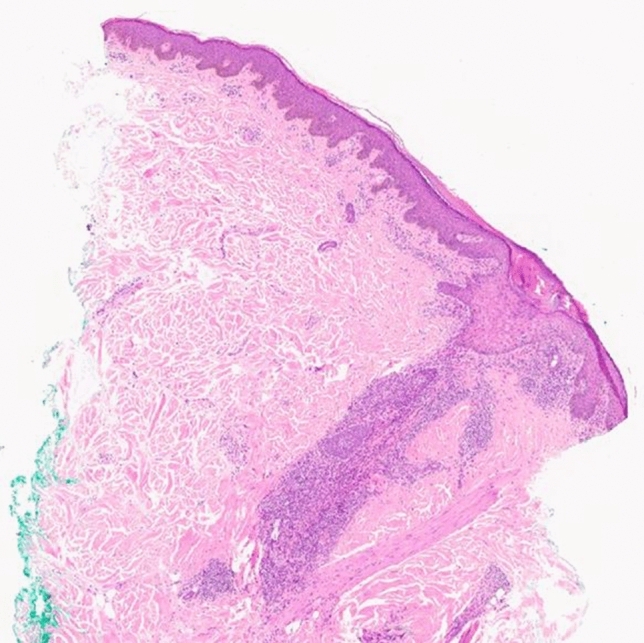
The histological findings of the left cheek showed an intact epidermis. In the dermis were perifollicular and peri-adnexal inflammatory infiltrates with eosinophilic granulocytes. Additional staining (PAS, Giemsa, Ziehl Neelsen) were negative. 2× magnification, haematoxylin and eosin staining

**Fig. 4:**
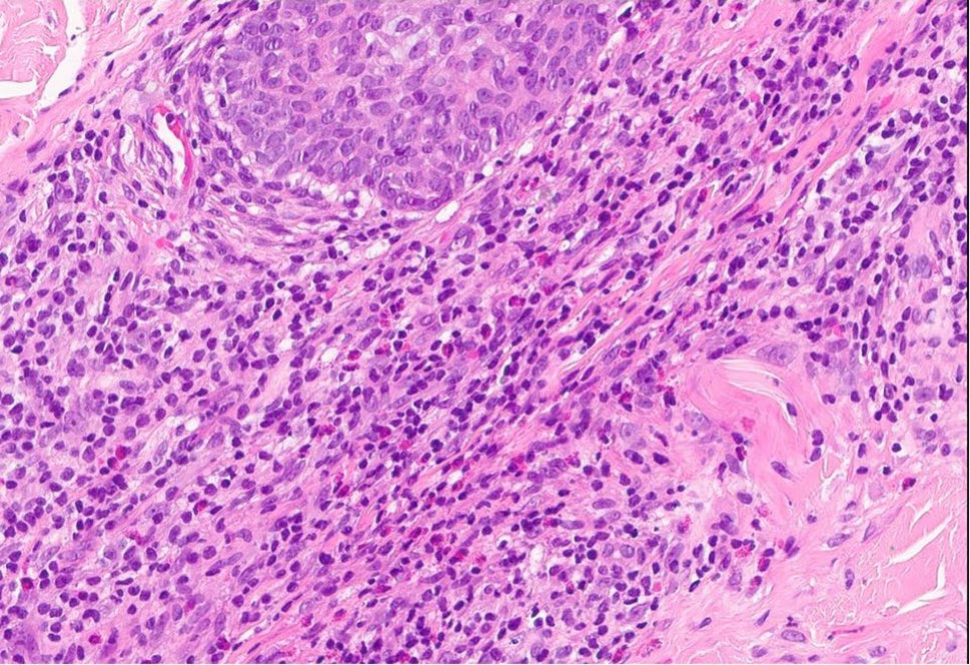
20× magnification, haematoxylin and eosin staining of the same histological site as in Fig. 3

EPF is a chronic, recurrent dermatosis, of unknown etiology, which is histologically characterized by folliculotropic inflammatory infiltrates with admixed eosinophils in the dermis [1, 2]. Seborrheic areas are preferentially affected [2]. Clinical findings include pruritic papules, anular plaques and pustules [2]. EF in women may predominantly affect the face and mimic acne excoriée des jeunes filles, whereas in men, it affects more commonly the trunk [3]. It is divided in three forms: the classical EFP, the infantile and the immunosuppression-associated IS-EPF [1, 2, 4]. IS-EPF is associated with HIV Infection (IS-EPF/HIV) or malignancies [2, 5]. HIV-EF may be distinguished from EPF depending on several clinical and laboratory features. In HIV-EF, patients suffer on unremitting pruritus, palms and soles are spared and leucopenia is present. In contrast, fewer than 50% of the patients with EPF report pruritus, 20% have palm and sole involvement and most have leukocytosis [6]. Treatment of IS-EPF/HIV can be challenging [7]. First step in untreated HIV-positive patients with EPF is the initiation of combined antiretroviral therapy (HAART). Topical therapies include tacrolimus and steroids. UVB radiation can be used additionally. In case of persistence of the lesions topical ketoconazole, metronidazole, permethrin and systemic treatments, such as indomethacin, tetracyclines and itraconazole are further described as treatment options [7].

Our patient is being treated locally with Pimecrolimus 1% cream in combination with HAART. Under therapy had the patient a minimal improvement of the disease. With the recovery of the immune system and the reduction of the virus load, a significant improvement of the skin condition is expected.

Multiple, disseminated papules and post-inflammatory hyperpigmentations on the face and back of the patient, and on the back, multiple excoriations (Table 1).

**Table 1:** Prevalence of the most common differential diagnoses of EPF

Skin disease	Prevalence	Age
Acne vulgaris	70–80%	Adolescents
	54%*	> 25 years old
Rosacea	22%*	> 30 years old
Bacterial folliculitis	Not applicable	In every age
Dermatomycosis	6,8%	In every age
Seborrheic dermatitis	3–10%	In every age
Cutaneous T-cell lymphoma	Rare	> 40 years old
Follicular mucinosis	Rare	In every age, depending on type

^*^Variable depending on ethnicity

Left cheek: The histologic findings showed an intact epidermis. In the dermis were perifollicular and peri-adnexal inflammatory infiltrates with eosinophilic granulocytes. Additional stainings (PAS, Giemsa, Ziehl Neelsen) were negative. Image 3: 2 × magnification, haematoxylin and eosin staining. Image 4: 20 × magnification, haematoxylin and eosin staining.
